# Interaction between grasping and articulation: How vowel and consonant pronunciation influences precision and power grip responses

**DOI:** 10.1371/journal.pone.0265651

**Published:** 2022-03-22

**Authors:** Lari Vainio, Martti Vainio

**Affiliations:** 1 Phonetics and Speech Synthesis Research Group, Department of Digital Humanities, University of Helsinki, Helsinki, Finland; 2 Perception, Action & Cognition Research Group, Department of Psychology and Logopedics, Faculty of Medicine, University of Helsinki, Helsinki, Finland; University of Birmingham, UNITED KINGDOM

## Abstract

Grasping and mouth movements have been proposed to be integrated anatomically, functionally and evolutionarily. In line with this, we have shown that there is a systematic interaction between particular speech units and grip performance. For example, when the task requires pronouncing a speech unit simultaneously with grasp response, the speech units [i] and [t] are associated with relatively rapid and accurate precision grip responses, while [ɑ] and [k] are associated with power grip responses. This study is aimed at complementing the picture about which vowels and consonants are associated with these grasp types. The study validated our view that the high-front vowels and the alveolar consonants are associated with precision grip responses, while low and high-back vowels as well as velar consonants or those whose articulation involves the lowering of the tongue body are associated with power grip responses. This paper also proposes that one reason why small/large concepts are associated with specific speech sounds in the sound-magnitude symbolism is because articulation of these sounds is programmed within the overlapping mechanisms of precision or power grasping.

## Introduction

Hand movements are connected to mouth movements anatomically and functionally. For instance, monkey F5 premotor cortex includes neurons that are involved in shared programming of hand and mouth grasping [1]. When this premotor region of a monkey is electrically stimulated, the monkey executes involuntary hand-to-mouth actions in which a grip is closed and the mouth is opened while the hand is brought to the mouth–most likely reflecting connections between the two effectors operating together for eating behavior [2, 3].

In humans, the connection between hand and mouth motor processes manifests also itself in behavior that is not directly related to eating. For example, Darwin in 1872 [4] noted that when people are cutting something with scissors, they often involuntarily clench and unclench their jaws. A similar phenomenon is observed in children when they produce sympathetic mouth movements, such as tongue protrusions, in imitative synchrony with hand movements [5]. In line with these findings, it has been shown that, when participants are required to grasp an object and simultaneously pronounce a meaningless syllable, the more the object’s size requires finger opening for the grasp, the more the participants open their lips during vocalization [6]. Correspondingly, when participants reach to grasp objects while pronouncing a low (/ɑ/) or a high (/i/) vowel, the finger opening of the grasp increases with the low vowel in comparison to the high vowel [7].

The behavioral evidence mentioned above is in line with the mouth-hand mimicry theories, according to which people have a natural tendency to mimic their own manipulative hand actions with the articulators, which in turn manifests itself in systematic interactions between speech sounds and hand gestures [8–10]. These views are tightly related to gestural theories of language evolution assuming that gesturing of the protolanguage descended from action capabilities that were not communicative to begin with, such as grasping and other manipulatory activities [10, 11]. Many of these theories underline the evolutionary role of combining or replacing these manual gesture actions by imitating these manual signs with oral actions [12, 13]. Over time, these orofacial gestures might have begun to combine the orofacial gestures with phonatory sounds, leading to modern language [14]. Even those theories that do not agree that initially communication occurred entirely using gestures, to large extent, emphasize the audio-visual nature of communication accepting that gestures played a core role in early protolanguage [15, 16]. For instance, Falk’s [17] perspective of language evolution emphasizes iconic prosody and gestural elements of communication as a key substrate for initially communicating meaning to other individuals. Similarly, Żywiczyński, Wacewicz and Lister [18] propose that the first communicative signs were mimetic and primarily iconic. In line with this, Imai and Kita [19] proposed a special role for iconicity in language evolution. Their account holds that iconic gestures and iconic utterances enabled initial communication about events and object properties in the external world. Over time, iconicity of vocal and gestural signs was to some extent–but not entirely–replaced by non-iconic vocal signs.

Following these views, Ramachandran and Hubbard [20] have, for example, provided a concrete example of how articulatory organs might mimic hand gestures in speech. They proposed that mouth shape for pronouncing words that refer to smallness (e.g., “petite” and “teeny”) sympathetically mimic the small precision grip gesture made by opposing thumb and index finger. Correspondingly, it has been shown that gestures that reproduce the precision grip in different ways seem enable iconic gesturing of different aspects of the referent object such as height and shape [21]. While Ramachandran and Hubbard’s proposal links symbolic precision grip gesture with an articulatory gesture, we have shown empirically that the non-symbolic precision grip action is connected to pronunciation of, for example, the vowel [i] and the consonant [t] [22]. This finding is in the focus of the present study, and hence it is unwrapped in the next section.

### The grip-sound effect

The terminal phase of different kinds of grasps can, in general, be divided into precision and power grips [23]. The precision grip has evolved in primates for grasping and manipulating small objects so that the object is pinched between the tips of the index finger and thumb. In contrast, the power grip has evolved for grasping larger objects using a clamp formed by the partly flexed fingers and the palm. These grip types have their own functional, neural and developmental characteristics [24–26].

Given this clear distinction between the two grip types, and the tight connection between mouth movements and grasping [1, 6], we hypothesized that if the mouth-hand mimicry theories are valid, certain articulatory gestures might be more closely associated with one of these grips than with the other. In order to investigate this assumption, we developed a dual-action procedure [22] in which the participants were visually presented with a meaningless syllable (e.g., [ti] or [kɑ]) in green or blue color. One of these syllables was hypothesized to be congruent with the precision grip (e.g., [ti]) and other with the power grip (e.g., [kɑ]). They were required to pronounce this syllable as fast as possible while simultaneously performing either a precision or a power grip response according to the color by squeezing either the precision or power grip response device in their hand.

This task revealed that the execution of the precision grip was facilitated when the participants had to pronounce the syllable [ti] in the [ti]-[kɑ] block, [te] in the [te]-[ke] block and [hi] in the [hi]-[hɑ] block. In contrast, the execution of the power grip was facilitated when the participants had to pronounce the syllable [kɑ] in the [ti]-[kɑ] block, [ke] in the [te]-[ke] block and [hɑ] in the [hi]-[hɑ] block [22, 27, 28]. That is, the facilitated precision grip performance was associated with the pronunciation of the unrounded high-front vowel [i] and the voiceless alveolar stop [t], while the facilitation of the power grip response was associated with the pronunciation of the unrounded-low-back vowel [ɑ] and the voiceless velar stop [k]. Importantly, the grip-sound effect can be observed in speakers of different language families (i.e., a Finno-Ugric/Uralic language, and a Slavic/Indo-European language) [29].

Complying with the mouth-hand mimicry accounts, we have proposed that the grip-sound effect reflects overlapped action planning of analogous action goals for separate but closely connected mouth and hand movements: a hand shape for grasping and a vocal tract shape for articulation [22]. In fact, the primary aim of the study is to explore this articulatory hypothesis of the grip-sound effect using new set of vowels and consonants in the design. This perspective is consistent with observations showing that two simultaneously performed actions, performed with separate effectors (e.g., the left and right hand, or a hand and mouth), accompany each other if they share some spatial characteristics (e.g., opening/closing or movement direction) [6, 30, 31]. Hence, regarding vowel production, the articulatory gesture for producing the high-front vowel [i] is an articulatory counterpart for the precision grip as it is analogously formed by a narrow mouth shape in which the tongue tip is pushed into a high-anterior position. In contrast, the articulation of the low-back vowel [ɑ], involving relatively great vocal tract opening, is associated with the power grasping because the power grasp is analogously used to grasp large objects with a relatively great aperture between the thumb and rest of the fingers.

Regarding consonants, the similarities between the different grips and the articulation of the corresponding speech sounds are not as intuitive as the similarities between the different grips and the articulation of the corresponding vowels. However, we have previously proposed [22] that similarly to vowels, the grip-sound effect related to consonants might be also based on analogies between the shape of the articulatory gesture and the corresponding grip type. The precision grip employs the tips of the thumb and the index finger forming a hand into a narrow pincer shape. This grip type is associated with the articulatory gesture of the consonant [t] as it is analogously produced by bringing the tip of the tongue into contact with the opposing surface of the alveolar ridge and back of upper-central incisors–similarly to the vowel [i]–forming the front of the oral cavity into a narrow pincer shape. In contrast, the articulatory gesture that is produced by moving the back of the tongue body against the velum (e.g., [k]) is associated with the power grip as this grip is analogously formed by moving intermediate and proximal components of all fingers (i.e., finger bodies instead of fingertips as in the precision grip) against the opposing palmar surface of the hand.

We have also previously shown that the [r] (voiced alveolar trill) (in the [re]-[ke] block) is associated with the precision grip assumably because similarly to [t] it is produced by fronting the tip of the tongue [32]. In addition, [u] (rounded high-back) (in the [hu]-[hɑ] block) has been shown to be associated with the precision grip. The vowel [u] was proposed to mimic the precision grip because when producing [u], the lips form a small, protruded shape [22]. This perspective is in line with the finding showing increased EMG responses of the orbicularis oris muscles–involved in producing articulations requiring lip protrusion–during execution of precision grasping [33]. Furthermore, [m] (voiced bilabial nasal) (in the [pe]-[me] block) has been shown to be associated with the power grip [22]. The association between [m] and the power grip might be linked to the fact that, similarly to low-back vowels, it is produced with enlarged oral cavity, which provides a resonance chamber the nasal sound.

### The research questions of the present study

Sound symbolism refers to the non-arbitrary associations that exist between phonetic and/or articulatory properties of speech sounds and their meaning [34]. Sound symbolism is highly relevant research topic for cognitive scientists, linguistics, evolutionary psychologists, and theoretical phonologists [19, 35, 36]. It has been proposed that many sound-symbolic phenomena can be based on the articulatory and/or acoustic properties of speech sounds [37, 38]. We have previously proposed that the grip-sound effect and the sound-magnitude symbolism effect might be–to some degree–based on the same representational mechanisms [39]. The sound-magnitude symbolism refers to systematic mental associations between specific speech sounds and magnitude concepts [40]. Most commonly, the high-front vowel [i] is associated with small magnitudes [41–43]. Additionally, more rarely, close-mid-front vowel [e] is also linked to small magnitudes [44, 45]. In contrast, low and/or back vowels, such as [ɑ], [o], [u] and [æ] are associated with large magnitudes [43, 45–47].

The picture is more mixed and complex for consonants. The general rule of thumb is that voiced consonants (e.g., [m], [l], [g] and [b]) are more likely to be associated with large concepts while voiceless consonants (e.g., [t] and [s]) are associated with small concepts [43, 48–51]. Indeed, it has been shown, for instance, that the voiceless alveolar plosive [t] [36] and voiceless palato-alveolar affricate [ʧ] [42] are associated with small concepts. However, Newman’s [43] study suggests that voiced consonants such as [d] can be also associated with small magnitudes. Furthermore, Taylor [52] has suggested that the consonant [k] might be associated with large magnitudes even though it is a voiceless consonant. For example, according to Taylor [52], Newman [43] may have missed that words referring to large concepts frequently contain the velar stops /g/ and /k/ (e.g., *gargantuan*, *glaring*, *great*, *gross*, *colossus*, *cargo*, *comprehensive*, *corporation*, *corpulence*).

Our account assumes that the sound-magnitude symbolism can be based on acoustic and gestural aspects of vocalization. Regarding the acoustic origin of the phenomenon [53, 54], because small things (e.g., objects and animals) tend to produce relatively high-frequency sounds, speech sounds with higher spectral components − such as [i], which yields relatively high fundamental frequency [55, 56] − are associated with small concepts. Regarding our gestural origin of the phenomenon [39], small concepts and large concepts are associated with certain vowels and consonants because there is functional overlap between representations of articulatory gestures and grasp types. For example, small things are associated with speech sounds that are produced by narrowing a vocal tract, such as [i], or by raising the tongue tip into contact with the alveolar ridge, such as [t], because the same mechanisms that are involved in programming these articulatory gestures are also involved, to some extent, in programming the precision grip (i.e., a grasp type that is used to grasp small things).

One of the goals of the present study is to investigate whether the grip-sound effect associates the precision and power grip responses with the same vowels/consonants as that found between speech sounds and small/large sizes in sound-magnitude symbolism research. If the same vowels/consonants were associated with the precision and power grips as that found in the sound symbolism literature in relation to small and large concepts, respectively, it would support the view that the grip-sound effect and the sound-magnitude symbolism can, indeed, be based on the same representational mechanisms. At the same time, the study aims to complement the picture about which vowels and consonants are associated with precision and power grips. As a result of this, we hope to present a clearer picture of mechanisms behind the grip-sound effect, which either validates or forces an update of our previous views concerning how these grip types are connected to particular speech sounds.

Finally, this study aims to clarify the methodological aspects linked to the grip-sound effect. Previous grip-sound studies have recruited the “Type 3 Ensembles” (T3E) version of the stimulus-response (S-R) compatibility task [57] that has commonly been used to investigate, for example, the spatial S-R compatibility effect [58]. In this task, the S-R ensemble of a single experimental block consists of two opponent responses (e.g., the precision and power response) and two opponent stimulus types (e.g., [ti] and [kɑ]). The S-R ensemble is formed so that one of the stimuli (e.g., [ti]) provides a (hypothetically) maximally compatible match to one of the responses (e.g., precision grip) and a maximally incompatible match to the other response (e.g., power grip). Similarly, the other stimulus (e.g., [kɑ]) provides a maximally compatible match to the other response (e.g., power grip) and a maximally incompatible match to the other response (e.g., precision grip). The T3E task can be assumed to enable and strengthen an implicit allocation of both opponent responses to a single, but different, repetitively and frequently presented stimulus alternative [57]. This might heighten the capability of a single stimulus (e.g., [ti]) to automatically facilitate congruent responses (e.g., articulating [ti] and producing the precision grip) and suppress incongruent responses (e.g., articulating [kɑ] and producing the power grip).

Furthermore, another reason for applying this blocked T3E design in our previous studies was that S-R effects have often been found to be based on relative rather than absolute differences between the stimuli that trigger the effect. For example, the Simon effect (i.e., compatibility effect between the left-right target location and the responding hand; [58]) is observed in relation to the left-right location of the target relative to the initial fixation stimulus rather than absolute left-right location of the target in the visual field [59]. Similarly, the frequently observed perceptual cross-modal congruency effect between the pitch of an auditory stimuli and the size of a visual target [60] is based on relative pitch differences between two auditory stimuli rather than their absolute frequencies [61]. Therefore, it could be assumed that the blocked design consisting of two opponent stimuli (e.g., [ti] and [kɑ]) would highlight the relative difference between the stimuli providing the most optimal ground for observing the grip-sound effect.

Thus, the present study asks whether the grip-sound effect can be observed even when a single experimental block consists of several vowel/consonant stimuli, and whether the effect is boosted when the T3E blocked method is recruited. Given the robustness of the grip-sound effect, it is likely that the paired setting is not in fact required, and that a mixed design with many different speech units presented within the same block would also reveal the effect. Therefore, different experiments of the current study used blocked (Experiment 1) and mixed (Experiments 2) designs.

### Experiment 1

Experiment 1 recruits the T3E blocked design similarly to our previous studies [22, 32]. In each trial, participants are thus presented a vowel or consonant in green or blue color and asked to pronounce it as fast as possible while simultaneously performing either precision of power grip response according to the color. Our main interest was on measuring manual RTs and response accuracy (i.e., errors) in relation to interactions between different speech units and grip types. As one of the research questions, Experiment 1 investigates whether the same vowels/consonants that are typically associated with large ([o], [u], [æ], [l], [m], [k]) and small ([i], [t], [d]) magnitudes [40–52] are also associated with the precision/power grip responses. Our previous studies provide very limited evidence for this perspective because those studies have used only small set of vowels/consonants. The present study uses a larger set of vowels/consonants in order to explore this hypothesis. In order to construct the T3E design to the study, these vowels/consonants of interest were presented in separate blocks that also contained a speech unit ([i] or [t]) that has been previously established to be associated with the precision grip. Therefore, the study consisted of blocks [i]-[o], [i]-[u], [i]-[æ], [t]-[l] and [t]-[m]. In addition, we were particularly interested in whether [d] is associated with the precision grip because, similarly to [t] that has been previously associated with the precision grip [22], it is an alveolar stop, and because sound symbolism research has linked it to small magnitudes [43]. Hence, [d] was presented in a block that also contained the speech unit [k] that has been previously associated with the power grip, forming a block of [d]-[k]. Finally, in order to minimize a fatigue effect, Experiment 1 is comprised of three separate studies from which one explored the blocks [i]-[o] and [i]-[u], one explored the block [i]-[æ], and one explored the blocks [t]-[l], [t]-[m] and [d]-[k].

## Methods

### Participants

Sixteen naïve volunteers participated in Experiments 1a (19–37 years of age; mean age = 24.9 years; 5 males), and 1b (19–48 years of age; mean age = 24.9 years; 3 males). Seventeen volunteers participated in Experiment 1c (19–36 years of age; mean age = 24.5 years; 3 males). The selection of number of participants was based on our previous similar investigation of the grip-sound effect, which has shown significant (η_p_^2^ = .696) effects using twelve participants [22]. All participants were right-handed and had a normal or corrected- to-normal vision. At this point, it should be mentioned that as all the experiments reported in this paper were conducted with native Finnish participants, only the phonemes allowed by Finnish phonology and phonotactics were used. All of the participants were naïve as to the purpose of the study; they appeared entirely unaware of the purpose of the study and the nature of the investigated effect when this information was asked of them after the experiment.

### Ethics statement

We obtained a written informed consent from all participants. The study was approved by the Ethical Review Board in Humanities and Social and Behavioural Sciences at the University of Helsinki.

### Apparatus, stimuli and procedure

Each participant sat in a dimly lit room with his or her head 60 cm in front of a 19-inch CRT monitor (screen refresh rate: 100 Hz; screen resolution: 1280 x 1024). There were two response devices, each equipped with an inlaid micro-switch: the precision grip device (1×1×0.7 cm) and the power grip device (11 cm long, 3.2 cm diameter). The responding hand was resting on a table, in front of the monitor, so that the axis of elongation of the power grip device was rotated vertically. As the switches were depressed in each device, there was noticeable tactile feedback.

The stimuli of Experiment 1a consisted of two vowel pairs (I-O & I-U), the stimuli of Experiment 1b consisted of one vowel pair (I-Ä) (notice that Ä is pronounced as [æ]), and the stimuli of Experiment 1c consisted of three consonant-vowel syllable pairs (TE-LE, TE-ME & DE-KE). As such, these ensembles containing a pair of speech units were treated as experimental blocks. The order of the blocks and the speech units within the blocks was randomized. The participants were allowed to have a short break before each new block. Although Experiment 1c investigated interaction between consonants and grip types, similarly to the original study, the consonants were coupled with the vowel [e] because it is difficult to pronounce consonants alone. The vowel [e] was selected because it is unrounded and relatively neutral vowel in the Finnish language in terms of tongue position: that is, it is not as front as [i] or [y] or as back as [ɑ], [o] or [u]. In addition, it is not as closed as [i] or [y] or as open as [ɑ] or [æ].

The target was a single speech unit of each block. The targets were presented centrally on the monitor on a light-grey background. They were presented in the uppercase-form of the Consolas-font (font size 72) in order to avoid any large size differences between the stimuli. The trial started with a presentation of a central fixation cross (1° x 1°), which was displayed for 400 ms. A blank screen was displayed for 200 ms after the offset of the cross. Then the target was presented for 1500 ms or until a response was made. Finally, a blank screen was displayed for 1700 ms after the offset of the target.

The participants held the grip devices in their right-hand. The target stimuli were presented in green or blue color. The order of the colors was randomized. The participants were required to respond as fast and accurately as possible according to the color. Half of the participants were asked to respond by pressing the precision grip device if the stimulus was green and with the power grip device if it was blue. The other half responded in the opposite response-to-color mapping. In addition to making the grip responses, the participants were asked to pronounce the vowel/syllable at the same time as they gave the grip response. It was emphasized that the vowel/syllable should be uttered promptly in a natural spoken tone. Each participant was given as much practice as it took to perform the task fluently. This practice phase required on average 15 trials. The experiment was not started until the participant was able to perform accurate and fast manual and vocal responses simultaneously. Estimation of the adequate response speed, accuracy and simultaneity was based on the experimenter’s observation. In addition, there was a practice session of twenty trials at the beginning of each new block in which the participants were familiarized with speech units that had to be pronounced in the upcoming block. Erroneous manual responses were immediately followed by a short “beep” tone. The participants were allowed to have two short breaks within the experiment. In total, Experiment 1a lasted around 10 minutes and consisted of 200 trials [25 x 2 (block) x 2 (vowel) x 2 (grip)], Experiment 1b lasted around 6 minutes and consisted of 100 trials [25 x 2 (vowel) x 2 (grip)] and Experiment 1c lasted around 15 minutes and consisted of 264 trials [22 x 3 (block) x 2 (consonant) x 2 (grip)].

## Results

### Reaction times

Reaction times were measured from the onset of the target stimulus. In Experiment 1a, in total, 5.9% of the raw data were discarded from the RT analysis including 4.1% of trials containing errors, 0.1% of trials containing no-responses and 1.7% of trials in which the RTs were slower than 1000 ms. In Experiment 1b, in total, 3.5% of the raw data were discarded from the RT analysis including 1.9% of trials containing errors, 0.09% of trials containing no-responses and 1.5% of trials in which the RTs were slower than 1000 ms. In Experiment 1c, in total, 6.1% of the raw data were discarded from the RT analysis including 3.8% of trials containing errors, 0.09% of trials containing no-responses and 2.2% of trials in which the RTs were slower than 1000 ms.

The reaction time data of Experiments 1a, 1b and 1c (https://osf.io/aes9u/) were analyzed in a single linear mixed model analysis. The analysis treated Block (1 = [i]-[o], 2 = [i]-[u], 3 = [i]-[æ], 4 = [t]-[l], 5 = [t]-[m] or 6 = [d]-[k]), Speech unit (1 = precision grip related: Block1 = [i], Block2 = [i], Block3 = [i], Block4 = [t], Block5 = [t], Block6 = [d] or 2 = power grip related: Block1 = [o], Block2 = [u], Block3 = [æ], Block4 = [l], Block5 = [m], Block6 = [k]) and Grip (precision or power) as fixed factors and Subject as a random intercept. Selection of error covariance structure was based on Schwarz’s Bayesian information criterion (BIC). All tests of pairwise comparisons were carried out using Bonferroni correction for multiple comparisons. The analysis was carried out using SPSS software package (version 27).

After estimating the best-fitting error covariance structure (BIC = 109897.77), the analysis of reaction times revealed a significant main effect of Grip [F(1,41) = 18.54, p < .001]. Precision grip responses were made faster (M = 506 ms) than power grip responses (M = 528 ms) replicating our previous observations [22]. This difference can be attributed to the fact that the power grasp recruits more fingers that the precision grasp. In addition, the interaction between Speech unit and Grip [F(1,8648) = 295.29, p < .001] as well as Block, Speech unit and Grip [F(5,8648) = 6.72, p < .001] were significant.

**Fig 1:**
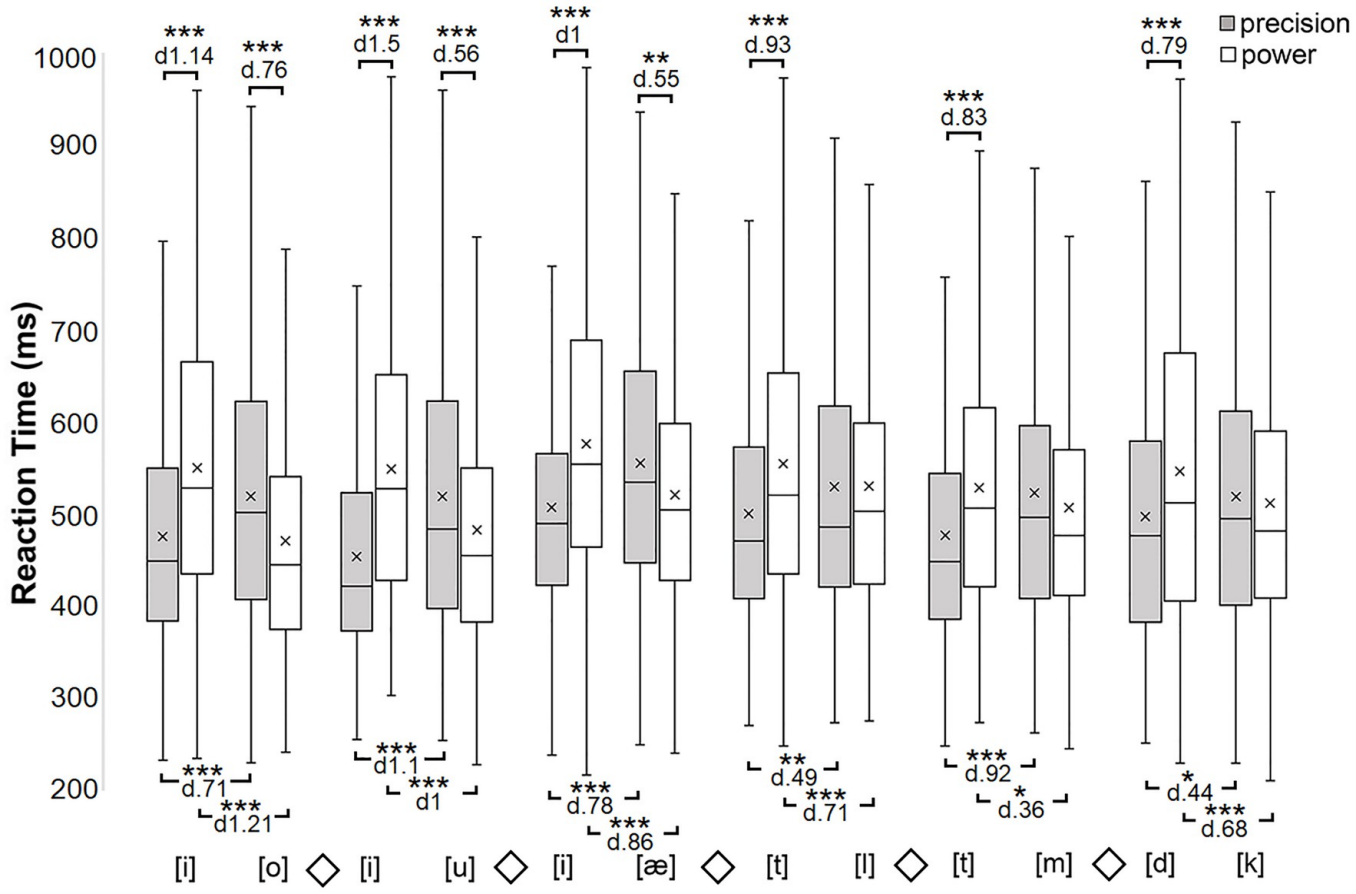
Box-plot of reaction times (ms = millisecond) for Experiment 1 (horizontal line inside the box = median; cross inside the box = mean; box = 25–75%; whiskers = scores outside the middle 50%). The box-plots show the distributions of RT (reaction time) values as a function of Block, Speech unit and Grip. Asterisks indicate statistically significant differences (***p < .001, **p < .01, *p < .05).

The pairwise comparison test showed that precision grip responses are made significantly faster than power grip responses when the pronounced speech unit is [i] (in Blocks 1, 2 and 3), [t] (in Blocks 4 and 5) and [d] (in Block 6). In contrast, power grip responses were made significantly faster than precision grip responses when the pronounced speech unit was [o], (Block 1) [u], (Block 2) and [æ] (Block 3). Correspondingly, precision grip responses were made significantly faster with [i] than with [o] (Block 1), [u] (Block 2) and [æ] (Block 3); with [t] than with [l] (Block 4) and [m] (Block 5); and with [d] than with [k] (Block 6). In contrast, power grip responses were made significantly faster with [o], [u] and [æ] than with [i] (Blocks 1–3); with [l] and [m] than with [t] (Blocks 4 & 5); with [k] than with [d] (Block 6). The p-values and effect sizes are presented for these interactions in Fig 1. These observations present that the speech units [i], [t] and [d] are associated with precision grip responses and [o], [u], [æ], [l], [m] and [k] are associated with power grip responses.

### Error rates

The percentage error rates were analyzed for Experiments 1a, 1b and 1c using a single linear mixed model analysis that similarly to the reaction time analysis treated Block, Speech unit and Grip as fixed factors and Subject as random intercept. One participant did not make any errors in Experiments 1a and 1c. All tests of pairwise comparisons were carried out using Bonferroni correction for multiple comparisons. The analysis (BIC = 2237.56) revealed a significant interaction between Speech unit and Grip [F(1,312) = 58.95, p < .001] as well as interaction between Block, Speech unit and Grip [F(5,312) = 3.21, p = .008]. The pairwise comparison test showed significant difference between precision and power grip responses, in Block 1, for the speech units of [i] (precision: M = 0.8%, power: M = 9.3%, mean difference = 8.5%, SE = 1.7, p < .001, d_z_ = 0.89) and [o] (precision: M = 4.5%, power: M = 1.1%, mean difference = 3.4%, SE = 1.7, p = .042, d_z_ = 0.36); In Block 2, for [i] (precision: M = 0.8%, power: M = 9.1%, mean difference = 8.3%, SE = 1.7, p < .001, d_z_ = 0.87) and [u] (precision: M = 5.9%, power: M = 1.9%, mean difference = 4%, SE = 1.7, p = .014, d_z_ = 0.42); In Block 3, for [i] (precision: M = 0.3%, power: M = 4.0%, mean difference = 3.7%, SE = 1.6, p = .023, d_z_ = 0.39); In Block 4, for [te] (precision: M = 1.7%, power: M = 5.1%, mean difference = 3.4%, SE = 1.6, p = .039, d_z_ = 0.36) and [le] (precision: M = 5.7%, power: M = 1.7%, mean difference = 4%, SE = 1.6, p = .016, d_z_ = 0.42); In Block 5, for [me] (precision: M = 5.7%, power: M = 1.1%, mean difference = 4.6%, SE = 1.6, p = .006, d_z_ = 0.49).

In addition, in Block 1, the participants made fewer errors when they were required to respond with the precision grip and the Speech unit was [i] (M = 0.8%) rather than [o] (M = 4.5%) (mean difference = 3.7%, SE 1.7 =, p = .029, d_z_ = 0.39), and when they were required to respond with the power grip and the Speech unit was [o] (M = 1.1%) rather than [i] (M = 9.3%) (mean difference = 8.2%, SE = 1.7, p < .001, d_z_ = 0.86). In Block 2, the participants made fewer errors when they were required to respond with the precision grip and the Speech unit was [i] (M = 0.8%) rather than [u] (M = 5.9%) (mean difference = 5.1%, SE 1.7 =, p = .029, d_z_ = 0.54), and when they were required to respond with the power grip and the Speech unit was [u] (M = 1.9%) rather than [i] (M = 9.1%) (mean difference = 8%, SE = 1.7, p < .001, d_z_ = 0.76). In Block 4, the participants made fewer errors when they were required to respond with the precision grip and the Speech unit was [te] (M = 1.7%) rather than [le] (M = 5.7%) (mean difference = 4%, SE 1.6 =, p = .016, d_z_ = 0.42), and when they were required to respond with the power grip and the Speech unit was [le] (M = 1.7%) rather than [te] (M = 5.1%) (mean difference = 3.4%, SE = 1.6, p = .039, d_z_ = 0.36). In Block 5, the participants made fewer errors when they were required to respond with the precision grip and the Speech unit was [te] (M = 1.1%) rather than [me] (M = 5.7%) (mean difference = 4.6%, SE = 1.6, p = .006, d_z_ = 0.42). These observations are mostly in line with the grip-size effect observed in reaction times with the exceptions of [æ], [d] and [k]. That is, [æ] and [k] were not linked to reduced error rates with power grip responses even though they were linked to response facilitation observed in reaction times when responses were performed with the power grip. Similarly, [d] was not linked to reduced error rates with precision grip responses even though it was linked to response facilitation observed in reaction times when responses were performed with the precision grip.

### Discussion

Similar to our previous studies, Experiment 1 recruited the T3E method to investigate which vowels/consonants provide the best matches to the precision and power grips. In reaction times, the results of Experiment 1, together with our previous observations, show a relative facilitation of precision grip responses in comparison to power grip responses when the stimulus calls for pronouncing the vowel [i]. Similarly, the consonants [t], [d] (the current study) and [r] [32] are stronger associated with faster precision grip than power grip responses. In contrast, the power grip response is relatively facilitated when the stimulus calls for pronouncing the [ɑ] [22], [u], [o] or [æ] (the current study). Similarly, power grip responses were performed particularly rapidly when the speech unit was [k] (in comparison to [d]), [l] (in comparison to [t]) or [m] (in comparison to [t]). Furthermore, the results of percentage error rates were mostly in line with the results of reaction times. In general, the participants made fewer errors when they were required to respond with the precision grip and the speech unit was [i] or [t], or when they were required to respond with the power grip and the speech unit was [o], [u], [l] or [m].

### Experiment 2

In Experiment 2 we used a similar behavioral paradigm that was also used in Experiment 1. In contrast to Experiment 1, however, Experiment 2 consisted of a wider variety of different consonants and vowels. The consonants and vowels were investigated in separate experiments. Experiment 2a included all vowels of the Finnish language ([ɑ], [e], [i], [o], [u], [y], [ɑ] & [ø]) from which [i] and [y] are high-front vowels, [e] and [ø] are mid-high-front vowels, [æ] is low-front vowel, [u] is high-back vowel, [o] is mid-low-back vowel and [ɑ] is low-back vowel. Consequently, if the effect is exclusively based on frontness, the vowels [i], [y], [e], [ø], [æ] should be matches for the precision grip and the vowels [u], [o] and [ɑ] for the power grip. In contrast, if the effect is exclusively based on openness, the vowels [i], [y] and [u] should be matches for the precision grip and the vowels [æ], [ɑ] for the power grip, while [o], [e] and [ø] might be placed somewhere between precision and power grip responses. Alternatively, it is possible that the effect operates within a combination of these two dimensions so that, for example, only high-front vowels are linked to the precision grip and low-back vowels are linked to the power grip.

Regarding consonants (Experiment 2b), in order to make the length of the experiment reasonable for the participants, we had to select only those consonants (i.e., [d], [k], [l], [m], [n], [p], [r], [s] & [t]) that were hypothetically most suitable for testing our primary research questions. Consonant can be distinguished by several phonetic features such as the manner of articulation (stops, fricatives, and nasals), the place of articulation (bilabial, alveolar and velar) and the phonation (voiced and voiceless). Our previous studies suggest that voicing does not seem to be an imperative feature in the grip-sound effect because the power grip is associated with velar and voiceless [k] and the precision grip is associated with alveolar and voiceless [t] and alveolar and voiced [r] [22, 32]. As such, we have proposed [22, 32] that the effect is largely based on the place of articulation.

Hence, our primary hypothesis focused on investigating this assumption. We hypothesized that if the effect is exclusively linked to the place of articulation (i.e., alveolar vs. velar), the coronal consonants ([d], [l], [n], [r], [s] and [t]) should be associated with the precision grip whereas the dorsal consonant [k] should alone be associated with the power grip. In addition, some consonants, such as [m], [l], [t] and [s], were selected because they are commonly associated with small ([t] and [s]) or large ([m] and [l]) concepts in the sound symbolism literature [36, 43, 48–52]. However, at this point it is fair to emphasize that sound-symbolic associations are newer clear-cut given that, for example, the consonant [m] is not only associated with large size but it can be also associated with roundness [62] as well as cuteness and softness (e.g., babies) [63], which in turn might also associate [m] with smallness. That is, in addition to exploring which articulation characteristics link a consonant to a specific grip type, Experiment 2 also investigates whether the same vowels/consonants that are often associated with small/large magnitude are correspondingly associated with the precision/power grip responses.

## Methods

### Participants

Eighteen naïve volunteers participated in Experiment 2a (18–51 years of age; mean age = 25.2 years; 5 males), and twenty volunteers participated in Experiment 2b (19–31 years of age; mean age = 23.4 years; 4 males). All participants were right-handed, native speakers of Finnish and had a normal or corrected- to-normal vision. All of the participants were naïve as to the purpose of the study; they appeared entirely unaware of the purpose of the study and the nature of the investigated effect when this information was asked of them after the experiment.

### Ethics statement

We obtained a written informed consent from all participants. The study was approved by the Ethical Review Board in Humanities and Social and Behavioural Sciences at the University of Helsinki.

### Apparatus, stimuli and procedure

The apparatus, stimuli and procedure were the same as those used in Experiment 1 with the following exceptions. In Experiment 2a, the target stimuli consisted of eight vowels (A, E, I, O, U, Y, Ä & Ö) (notice Ö is pronounced as [ø] and Ä is pronounced as [æ]) whereas in Experiment 2b, the target stimuli consisted of nine consonant-vowel syllables (DE, KE, LE, ME, NE, PE, RE, SE & TE). In contrast to Experiment 1, the stimuli were not blocked to stimuli pairs as in Experiment 1. That is, all vowels/consonants were presented in the same block. In total, Experiment 2a consisted of 480 trials lasting approximately for 25 minutes [30 x 8 (vowel) x 2 (grip)] and Experiment 2b consisted of 540 trials lasting approximately for 30 minutes [30 x 9 (consonant) x 2 (grip)].

## Results

### Reaction times

In Experiment 2a, in total, 2.9% of the raw data were discarded from the RT analysis including 2.3% of trials containing errors, 0.09% of trials containing no-responses and 0.5% of trials in which the RTs were slower than 1000 ms. In Experiment 2b, in total, 3.2% of the raw data were discarded from the RT analysis including 2.9% of trials containing errors, 0.07% of trials containing no-responses and 0.2% of trials in which the RTs were slower than 1000 ms. The data of two participants (one from both experiments) were removed from the analysis because of unacceptable number of errors (more than 10%).

The reaction time data of Experiments 2a and 2b (https://osf.io/aes9u/) were analyzed in a single linear mixed model analysis. The analysis treated Speech unit (eight vowels and nine consonants = 17) and Grip (precision, power = 2) as fixed factors and Subject as a random intercept. Selection of error covariance structure was based on Schwarz’s Bayesian information criterion (BIC). There was a slight positive skew (skewness = 1.4) for reaction times, which something that is very commonly observed in reaction time data. However, it has been proposed that there is no reason to transform response times when analyzing them using linear mixed model analysis [64], given that this analysis is very robust to violations of normality assumption [65]. All tests of pairwise comparisons were carried out using Bonferroni correction for multiple comparisons. The analysis was carried out using the SPSS software package (version 27).

**Fig 2:**
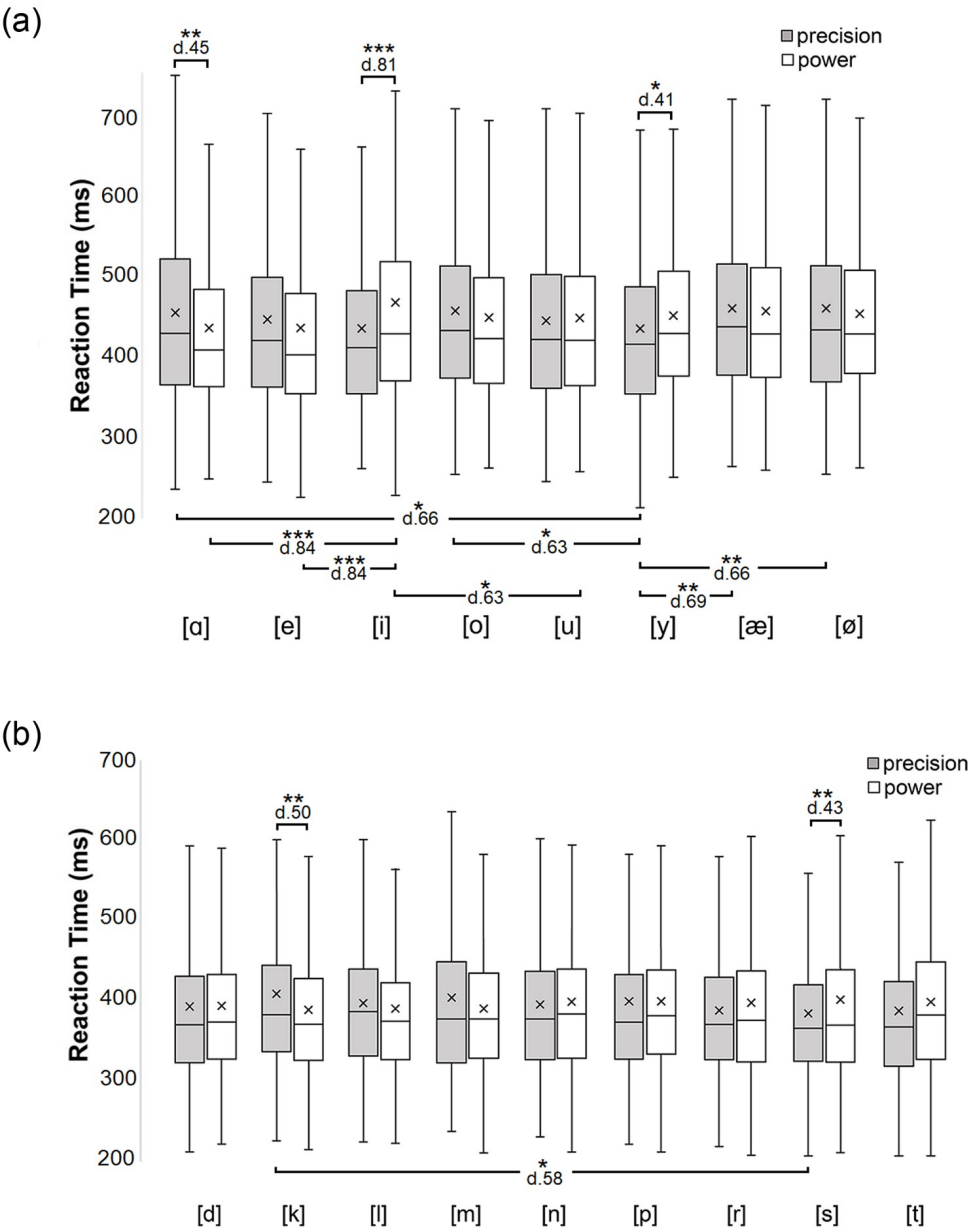
Box-plot of reaction times (ms = millisecond) for Experiment 2 (horizontal line inside the box = median; cross inside the box = mean; box = 25–75%; whiskers = scores outside the middle 50%). It shows the distributions of RT (reaction time) values as a function of Speech unit and Grip. Asterisks indicate statistically significant differences (***p < .001, **p < .01, *p < .05). The upper box-plots (Fig 2a) present the results for vowels and lower box-plots (Fig 2b) present results for consonants.

After estimating the best-fitting error covariance structure (BIC = 214004.67), the analysis of reaction times revealed a significant interaction between Speech unit and Grip [F(16,892) = 5.53, p < .001]. The pairwise comparison test showed that precision grip responses are significantly facilitated when the pronounced speech unit is [i], [y] and [s], while power grip responses are facilitated when the speech unit is [ɑ] and [k]. In addition, precision grip responses were performed particularly rapidly when the vowel was [y] in comparison to [ɑ], [o], [æ] or [ø]. Similarly, precision grip responses were performed particularly rapidly when the consonant was [s] in comparison to [k]. Moreover, power grip responses were performed particularly slowly when the vowel was [i] in comparison to [ɑ], [e] and [u]. The p-values and effect sizes are presented for these interactions in Fig 2a (for vowels) and 2b (for consonants). These observations present that precision grip responses are associated with the speech units [i], [y] and [s], while power grip responses are associated with the speech units [ɑ] and [k].

### Error rates

The percentage error rates were analyzed for Experiments 2a and 2b using a single linear mixed model analysis that similarly to the reaction time analysis treated Speech unit and Grip as fixed factors and Subject as random intercept. All tests of pairwise comparisons were carried out using Bonferroni correction for multiple comparisons. The analysis (BIC = 3158.90) revealed a significant interaction between Speech unit and Grip [F(16,297) = 4.24, p < .001]. The pairwise comparison test showed significant difference between precision and power grip responses for the speech units of [i] (precision: M = 1.6%, power: M = 5.3%, mean difference = 3.7%, SE = 1, p < .001, d_z_ = 0.54), [k] (precision: M = 4.7%, power: M = 0.5%, mean difference = 4.2%, SE = 1, p < .001, d_z_ = 0.56), [m] (precision: M = 4.9%, power: M = 1.9%, mean difference = 3 ms, SE = 1, p = .004, d_z_ = 0.4), [r] (precision: M = 1.9%, power: M = 4.4%, mean difference = 2.5 ms, SE = 1, p = .018, d_z_ = 0.34) and [t] (precision: M = 1.9%, power: M = 5.1%, mean difference = 2.5%, SE = 1, p = .002, d_z_ = 0.43). In addition, the participants made fewer errors when they were required to respond with the power grip and the vowel was [e] (M = 0.4%) rather than [i] (M = 5.3%, mean difference = 4.9%, SE = 1.0, p < .001, d_z_ = 0.79). Regarding consonants, the participants made fewer errors when they were required to respond with the power grip and the consonants was [k] (M = 0.5%) rather than [r] (M = 4.4%, mean difference = 3.9%, SE = 1.0, p = .022, d_z_ = 0.62) or [t] (M = 5.1%, mean difference = 4.6%, SE = 1.0, p = .001, d_z_ = 0.71), and when the consonant was [n] (M = 0.9%) rather than [t] (M = 5.1%, mean difference = 5%, SE = 1.0, p = .005, d_z_ = 0.66). As such, the analysis of error rates presents that the speech units [i], [r] and [t] are linked to precision grip responses, while [k], [m], [e] and [n] are associated with power grip responses.

The Table 1 presents the frontness, openness and roundedness dimensions of the vowels included in the current study. The “related grip” columns show how different vowels are linked to precision and power grip responses. In these columns, three horizontal lines mean that a given vowel was not included in the experiment and three crosses mean that a given vowel did not produce any association with the precision or power grip. The “SS-size” column presents the size (small vs. large) to which the given vowel has been linked in the previous research (references are provided in the main text).

**Table 1:** 

vowel	frontness	openness	roundedness	related grip (Ex1)	related grip (Ex2)	SS-size
[ɑ]	back	low	unrounded	---	power grip	large
[e]	front	mid-high	unrounded	---	_xxx_	small
[i]	front	high	unrounded	precis. grip	precis. grip	small
[o]	back	mid-high	rounded	power grip	_xxx_	large
[u]	back	high	rounded	power grip	_xxx_	large
[y]	front	high	rounded	---	precis. grip	unsolved
[æ]	front	low	unrounded	power grip	_xxx_	large
[ø]	front	mid-high	rounded	---	_xxx_	unsolved

The Table 2 presents PoA (place of articulation) and phonation (voiced vs. voiceless) dimensions of the consonants included in the current study. The “related grip” columns show how different consonants are linked to precision and power grip responses. In these columns, three horizontal lines mean that a given consonant was not included in the experiment and three crosses mean that a given consonant did not produce any association with the precision or power grip. The “SS-size” column presents the size (small vs. large) to which the given vowel has been linked in the previous research (references are provided in the main text).

**Table 2:** 

consonant	PoA	phonation	related grip (Ex1)	related grip (Ex2)	SS-size
[d]	alveolar	voiced	precision grip	_xxx_	small
[k]	velar	voiceless	power grip	power grip	large
[l]	alveolar	voiced	power grip	_xxx_	large
[m]	bilabial	voiced	power grip	_xxx_	large
[n]	alveolar	voiced	---	_xxx_	unsolved
[p]	bilabial	voiceless	---	_xxx_	small
[r]	alveolar	voiced	---	_xxx_	unsolved
[s]	alveolar	voiceless	---	precision grip	small
[t]	alveolar	voiceless	precision grip	_xxx_	small

### Discussion

In reaction times, precision grip responses were performed significantly faster than power grip responses when the speech unit was [i], [y] or [s], while power grip responses were performed significantly faster than precision grip responses when the speech unit was [ɑ] or [k]. These outcomes support our hypothesis that high-front vowels and alveolar consonants are relatively associated with the precision grip, while low-back vowels and velar consonants are more associated with the power grip. Corresponding interaction was observed in the results of percentage error rates in relation to [i], [e], [k], [m], [r], [t] and [n]. The participants made fewer errors with the precision grip than with the power grip when the speech unit was [i], [r] or [t]. In contrast, the participants made fewer errors with the power grip than with the precision grip when the speech unit was [k] or [m]. The fact that the participants made fewer errors with the power grip when the speech unit was [e] and [n] suggests that these speech units might be also associated with the power grip. However, given that these effects were not observed in reaction times, these interactions warrant for further investigation. However, some grip-sound interactions that were observed in Experiment 1, when the T3E blocked design was used, were not observed in Experiment 2. The fact that [o], [u] or [æ] from the vowels, and [d] or [l] from the consonants produced the grip-sound effect in Experiment 1 but not in Experiment 2 suggests that these sound units are rather weakly associated with their manual counterparts.

## General discussion

The results of this study supported our previous proposal that those vowels/consonants that are produced by pushing the tongue tip or blade (i.e., the area just behind the tongue tip) into a high-anterior position, in order to produce the speech sound, are stronger associated with precision grip responses than with power grip responses. In line with this view, our previous studies [22, 32] have shown that the high-front vowel [i] and the dental and alveolar consonants [t] and [r] are linked to the precision grip. Similarly, the present study showed that the high-front vowel [y] as well as the alveolar consonants [d] and [s] are also relatively associated with the precision grip. Therefore, it can be stated that the vowels that are high and front are relatively strongly associated with precision grip responses. Correspondingly, the consonants that are produced by moving the tongue tip or blade forward into a high-anterior position from its resting position are relatively strongly associated with precision grip responses.

We have proposed that the grip-sound effect reflects the connection between manual and articulatory processes in programming the goal-shape for separate but functionally integrated effector movements [22]. From this point of view, we propose that the vowels/consonants mentioned above are associated with the precision grasp because they are produced by a tongue tip-based and narrowed mouth shape that provides an oral counterpart to the pincer gesture. This oral action simultaneously provides a sensorimotor (proprioceptive) and somatosensory percept of a narrowing oral cavity at the front of the mouth, which from its side boosts associating these speech units with precision grip. Indeed, previous research suggests that in addition to perceptual cues related to hand movements [66], proprioception linked to hand movements might also contribute to sound symbolism [67].

Regarding power grip responses, this study supported our previous proposal that the vowels, which are low and/or back, are more associated with power grip responses than precision grip responses. That is, as a novel finding, this study showed that in addition to the vowel [ɑ] [22], the vowels [o], [u] and [æ] are also linked to facilitated power grip responses. That is, when the vowel is low ([æ]), back ([o] or [u]) or low-back ([ɑ]), it is relatively associated with the power grip. Furthermore, the current study replicated our previous observations concerning the consonant [k] [22] that was, also in this study, more associated with power grip responses in comparison to precision grip responses. As a novel finding, the results of Experiment 2 suggested that the lateral consonant [l] can be also linked to power grip responses.

The present study validated our prediction that the T3E blocked design facilitates observing the grip-sound effect. The vowels [o], [u] and [æ] as well as the consonants [d], [t], [m] and [l] produced the grip-sound effect when they were investigated in the T3E blocked design (Experiment 1), but not when they were investigated in the mixed design (Experiment 2). The only speech units that produced this effect in the mixed design were [i], [y] and [s] (in relation to the precision grip) as well as [ɑ] and [k] (in relation to the power grip). We propose that those speech units that produce the grip-sound effect even when they are presented in the mixed design provide the best (absolute) articulatory matches to the precision/power grip counterparts. In contrast, those speech units that produce the effect only in the T3E blocked design provide weaker (relative) articulatory matches to these grip types requiring that these speech units are presented so that the block consists of one optimal vocal match to both of the grip types.

In light of our articulation-grip hypothesis, we propose that the aspect which connects specific vowels and consonants to power grip responses is that articulating these speech sounds involve an active role of the back of the tongue (see Introduction for more detailed description of this view). Regarding the high-back vowels [u] and [o], the tongue back is raised, from its neutral position, upward and backward toward the dorsum. Similarly, [k] is produced by moving the back of the tongue toward the dorsum. Regarding the low vowel [æ], the tongue body is pulled downward, while in the low-back vowel [ɑ] the tongue root or body is pulled backward and downward toward the pharynx. Analogously to these low vowels, in the articulation of the consonants [m], which has been shown to be associated with the power grip [22], the tongue body is lowered in order to increase the oral cavity, which provides a resonance chamber for the nasal sound. The results of Experiment 1 − but not the results of Experiment 2 − replicated this effect with [m]. In addition to having the active role of the tongue back in these vowels/consonants, and consequently associating these speech sounds with the power grasp, articulation of the low vowels and the consonant [m] provides a somatosensory and sensorimotor (proprioceptive) percept of widening oral cavity. This aspect, from its side, also contributes to associating these speech sounds with the power grip that is analogously used to grasp large objects with a relatively great aperture between the thumb and rest of the fingers.

We propose that [l] is associated with the power grip, observed in Experiment 1, because its production involves an active role of the back of the tongue. In general, [l] can be articulated as a dark or clear version. Similar to velar consonants and high-back vowels, articulation of the dark [l] involves velarization so that the tongue back is raised toward the dorsum. In contrast, similarly to [m] and low-back vowels, articulation of the clear [l] is executed by retracting the back of the tongue. In Finnish, a darkness and clearness of [l] depends on a vowel context [68, 69]. For instance, the consonant appears to be clear rather than dark next to front vowel [i], while it is darker next to the [ɑ]. In the present study, [l] was pronounced next to the [e]. However, as far as we know, it is not known whether [l] is articulated as a purely dark or clear variant next to [e] in Finnish. Regardless of this, we can state that production of [l] involves an active role of the back of the tongue, making it suitable match to the power grip. Additionally, it is noteworthy that [l] is associated with power grip responses more than precision grip responses even though it is alveolar consonant similarly to [t], [d], [s] and [r]. This suggests that alveolar articulation does not inevitably link a consonant to the precision grip if articulation of the consonant also requires velarization or retracting the back of the tongue. Finally, it is possible that one reason why [l] was associated with the power grip in the block in which both speech targets were alveolar consonants (i.e., [t] and [l]) is because it is voiced. However, as stated earlier, based on our previous observations [22, 32] this is quite unlikely explanation for this finding.

An alternative explanation for the grip-sound effect is that those vowels/consonants that are associated with the precision or power grip have specific acoustic characteristics that somehow match them with these grip types. For instance, following Ohala’s frequency code hypothesis [53], already mentioned in Introduction, the grip-sound effect might be based on cross-modal mechanisms that link high frequency sounds with small concepts, assumably including the precision grip as it is used to grasp small things. Indeed, it has been shown that spectral peak is typically highest for the alveolar consonants–voiceless alveolar consonants in particular [70, 71]–that were also associated with precision grip responses in the present study. Concerning vowels, it is similarly possible, for example, that high-front vowels are associated with the precision grip because high vowels typically have higher fundamental frequency than low vowels [55, 72]. However, this explanation of the effect is not solid, because the current study shows that the high-back vowel [u] appears to be linked to power grip responses rather than precision grip responses. Furthermore, it has been shown [54] that fundamental frequency is not in fact the acoustic component that associates speech sounds with small/large magnitudes. Rather that study shows that the acoustic components that associated speech sounds with small and large magnitudes are the first and second formant. Increased first formant, which reflects tongue lowering, associates the speech sound with large magnitudes, while increased second formant, which reflects tongue fronting [73], associates the speech sound with small magnitudes [54]. Empirically, the question of whether the grip-sound effect is based on articulatory or acoustic aspects of speech is challenging because acoustic features are a consequence of articulation, and as a result, they cannot be separated from each other. Moreover, the acoustic and articulatory accounts of the grip-sound effect are not necessarily mutually exclusive, and it is possible that the acoustic *and* articulatory factors both contribute to the grip-sound effect.

The study did not replicate the originally observed interaction between [u] and precision grip [22]. Instead, here [u] was associated with the power grip in Experiment 1. This was, in fact, the only effect that was not in line with our previous observations concerning the grip-sound effect [22, 32]. The clearest difference between the present and the previous study was that contrary to the present study, in the previous study, the vowel [u] was articulated in the context of a consonant-vowel syllable with the glottal fricative [h] (i.e., [hu]) which has whisper like phonation similar to, for example, the aspiration portion of voiceless aspirated stops such as [p] and [t] in pre-vocalic position. Pronouncing this laryngeal fricative before the vowel [u] might have broken down the interaction effect that might otherwise associated this vowel with the power grip.

The results of this study mostly supported our views that the sound-magnitude effect and the grip-sound effect are based on overlapping mechanisms [39]. The same vowels and consonants that have been associated with large magnitudes in the sound symbolism literature ([ɑ], [o], [u], [æ], [k], [m] and [l]) also appear to be associated with the power grip responses. Similarly, the vowels and consonants that have been associated with small magnitudes ([i], [t], [s] and [d]) also appear to be associated with precision grip responses. As such, we propose that one reason small/large magnitudes are sound symbolically associated with specific vowels/consonant is because overlapping processes are involved in programming specific articulatory gestures and corresponding grasp types providing embodied representation for the abstract concepts of small and large.

The present study did not focus on exploring whether voiceness of a consonant contributes to the grip-size effect. The main reason for this was that our own previous studies suggest that the precision and power grips are not associated with voiceless and voiced consonants, respectively, given that the grip-size effect is observed when both consonants are voiceless (i.e., [t] and [k]) [22], and because the voiced consonant [r] is associated with the precision grip [32] even though according to the voiceness hypothesis it should be associated with the power grip. Moreover, as mentioned in Introduction, sound symbolism studies do not provide very clear outcomes concerning voiceness dimension of consonants. Some voiced consonants are associated with small sizes, and some are associated with large sizes [36, 38, 43, 48–52]. Nevertheless, this issue could be explored in the future studies, for example, by contrasting the voiced and voiceless consonants that are produced by similar tongue shapes (e.g., [g] and [k]).

In conclusion, the current study complements the picture about which vowels and consonants are associated with the precision and power grips. The results validated our previous views that the high-front vowels are more associated with precision than power grip responses, while high-back and low vowels are more associated with power than precision grip responses. Furthermore, these results supported our previous proposal that the alveolar consonants are relatively associated with precision grip responses, while the consonants that require velarization or whose articulation involves lowering of the tongue body are relatively associated with power grip responses. Finally, the current findings support the view that one of the reasons why large/small magnitudes are associated specific vowels/consonants is that articulation of these speech units is programmed within the overlapping mechanisms to precision or power grasping.

## Supporting information

S1 Data (XLSX)

S2 Data (XLSX)
